# Transactions of the American Medical Society

**Published:** 1867-10

**Authors:** 


Transactions of the American Medical Society.—We publish the following Circular from Dr. Wister for the information of our readers:

Philadelphia, September 28, 1867.

Dear Sir:—The Transactions of the American Medical Association, Vol. 18, are published, and now ready for delivery. Should you desire a copy, please remit five dollars to my address. As there are various methods by which the volume may be sent, inform me which you prefer. If by mail, please forward forty-four cents in post-office stamps, that your postage may be prepaid.

Very respectfully, Casper Wister.

Treasurer Am. Med. Association, 1303 Arch street.

The following volumes are for sale:—Proceedings of the Meeting of Organization, 50 cents. (Vols. 1, 2, 3, 4 and 6, are out of print.) Vols. 5, 7, 8 and 9, *if taken collectively*, $5 for the set. If singly, $2 apiece. Vols. 10, 11, 12, 13 and 14, $2 each. Vols. 15 and 16, $3 each. Vols. 17 and 18, $5 each.

